# *KIF18A* improves migration and invasion of colorectal cancer (CRC) cells through inhibiting *PTEN* signaling

**DOI:** 10.18632/aging.205027

**Published:** 2023-09-13

**Authors:** Yuan Liu, Ming Sun, Bin Zhang, Wenyan Zhao

**Affiliations:** 1 Department of General Surgery, Shengjing Hospital of China Medical University, Shenyang 110004, Liaoning, China; 2 Department of Urology, Shengjing Hospital of China Medical University, Shenyang 110004, Liaoning, China

**Keywords:** CRC, *KIF18A*, *PTEN*, *PI3K/Akt* signaling pathway, migration and invasion

## Abstract

Background: Kinesin family member 18A (*KIF18A*) is involved in the development of a variety of human malignancies. However, we have never known the influences of *KIF18A* on colorectal cancer (CRC). The study is designed to investigate the effect and molecular mechanism of KIF18A on the progression of colorectal cancer.

Methods: We have not only analyzed the database using GEO, but have examined the effect of *KIF18A* on the development of CRC by subcutaneous tumorigenesis in nude mice. HE staining was used to observe tumor size. Besides, we make use of Western blotting to monitor the expression of related proteins. In addition, the scratch wound assay and Transwell assay were conducted to detect the effect of *KIF18A* on the migration and invasion of CRC cells.

Results: The results of GEO database analysis suggested that KIF18A had a positive correlation with the growth of CRC. The results of subcutaneous tumorigenesis and HE staining in nude mice explained that *KIF18A* promoted the progression of CRC. Both scratch wound assay and Transwell indicated that the migration and invasion of CRC could be promoted by *KIF18A*. The results of Western blot illustrated that *KIF18A* could forward the migration and invasion of CRC cells, and inhibit *PTEN*, which promoted the activation of *PI3K/Akt* signaling pathway, thus bringing about the expression of *MMP2* and *MMP9*.

Conclusion: In conclusion, *KIF18A* can further the activation of *PI3K/Akt* signaling pathway by means of inhibiting *PTEN* transcription. Therefore, it is inferred that that *KIF18A* is a therapeutic target for CRC.

## INTRODUCTION

Based on the Global Cancer Statistics Report, colorectal cancer (CRC) is currently the third most common cancer and the second leading cause of cancer death [1]. About 35% of all cancer patients are defined as having metastatic disease [2], of which less than 20% patients can survive more than 5 years from the date of diagnosis [3]. The therapeutic targets in clinical practice only include DNA microsatellite instability and the changes in B-Raf proto-oncogene (*BRAF*) gene [4]. Investigating the histological characteristics of CRC is conducive to the research of the biological characteristics of tumors. Because colorectal cancer’s migration and invasion are one of main factors to the severity of the disease, exploring the molecular mechanisms of proliferation, migration and invasion of CRC cells will contribute to the development of targeted therapy [5].

*PTEN* (phosphatase and tensin homolog deleted on chromosome 10) belongs to a cancer suppressor gene (one of the protein phosphatases). Its catalytic phosphatase active center is structurally similar to phosphatase, so it is named *PTEN*.

*PTEN* can inhibit cell cycle progression and induce cell death to stimulate angiogenesis [6]. Promoting *PTEN* expression in CRC cell lines will stop the activation of the phosphatidylinositol-3-kinase (*PI3K*)/sinkserine/threonine kinase (*Akt*) signaling pathway, which affects CRC cell growth, promotes apoptosis, and improves sensitivity to targeted therapy, immunotherapy, and conventional therapy. Therefore, targeting drugs molecules in the *PTEN* signaling pathway may be another strategy to suppress tumor cells by inhibiting tumor activity [7, 8]. The study of oncogenes or suppressor genes crossing with *PTEN* will facilitate drug discovery and interpretation of chemotherapy resistance mechanisms.

Recombinant Kinesin Family, Member 18A (*KIF18A*) belongs to Recombinant Kinesin Family [9], which is highly expressed in tumors and associated with poor prognosis [10, 11]. It is a necessity of cell division and plays a role by regulating microtubule dynamics and chromosome aggregation [12]. In lung adenocarcinoma and prostate cancer, overexpression of *KIF18A* can motivate cell proliferation and suppress apoptosis. Furthermore, it is associated with tumor development [13, 14], and involved in invasion and metastasis [15]. The study focused on the role of KIF18A in colorectal cancer cell progression, migration and invasion, along with the pathways that KIF18A may affect. What’s more, it offered a new target and therapeutic strategy for the treatment of CRC.

## METHODS

### Cell culture

SW480 and HT29 (Human CRC cell lines) were bought from Procell (Wuhan, China) and they were cultured in DMEM with 10% fetal bovine serum and 1% penicillin/streptomycin. The cells were placed into a humid atmosphere of 37°C and with 5% carbon dioxide while the *PTEN* inhibitor SF1670 (MCE) was used to inhibit *PTEN* expression at a concentration of 10 μm.

### Transcriptome sequencing analysis

SW480 cells (1 × 10^6^) were put into each well of a 6-well plate. After 24 hours, the original medium was discarded, and the medium containing Rh3 (50 μg/mL) was added into the plate for another 24 hours. Then, the medium was removed, and the cells were rinsed with PBS, lysed and preserved with Trizol. GENEWIZ Co. (Beijing, China) carried on RNA purification, library preparation and RNA sequencing. Other steps were as follow: All RNA was extracted with Trizol; The libraries science was set by means of the NEBNext^®^ Ultra™ RNA Library Prep Kit for Illumina^®^ (NEB, Ipswich, MA, USA), and library preparations were sequenced on the Illumina Novaseq platform. The majority of the data of this research was analyzed and visualized by R packages. FastQC was used to check the quality of the reads. The adapter was cut out using Cutadapt (version 1.9.1) and mapped to the Ensembl human reference genome (EnsemblGRCh37 release 98) through Hisat2 (v2.0.2) aligner software. Differentially expressed genes (DEGs) were identified utilizing the R package, EdgeR. This package employs a negative binomial distribution model to account for both biological and technical variability inherent in RNA-seq data. Normalization of the raw read counts for each gene was achieved using the Trimmed Mean of M-values (TMM) method, which can adjust for the variances in library size and RNA composition. To define upregulation and downregulation, the log2 fold change (log2FC) for each gene was calculated. Genes exhibiting a log2FC greater than 1 were classified as upregulated, whereas those with a log2FC less than -1 were categorized as downregulated.

For statistical analysis, we employed the exact test provided by EdgeR, which is predicated on the negative binomial distribution. This test facilitated the comparison of gene expression differences between the experimental and control groups. To adjust for multiple testing, we utilized the Benjamini-Hochberg procedure, and genes with an adjusted *p*-value less than 0.05 were deemed significantly differentially expressed. The quantification of DEG expressions was determined using the counts per million (CPM) method, which can normalize gene counts based on the total number of reads in each sample, thereby enabling a fair comparison of gene expression levels across different samples. Visualization of these results was accomplished by generating a heatmap using the R tool. Lastly, GSEA version 4.1.0 was employed to execute and visualize Gene Ontology (GO) and KEGG GSEA queries.

### Cell infection

The targeting sequences for *KIF18A* (5′-GACUCAGACUCCAACGAAUTT-3′) was mounted on a lentiviral vector, respectively. A scrambled shRNA lentiviral vector was used as a negative control. To construct the overexpression vectors, the coding sequences of *KIF18A* were linked into a lentiviral vector driven by the U6 promoter. HT29 and SW480 Cells were transfected with the shRNA and overexpression recombinant lentiviral vectors in accordance with the instructions [13]. Then SW480 and HT29 cells were divided into four groups including sh-control group, sh-RNA *KIF18A* group, control group and *KIF18A*-OE group respectively.

### Experiments of subcutaneous tumorigenesis in nude mice

Five-week-old female BALB c nude mice were purchased from Nanjing Biomedical Research Institute of Nanjing University (Nanjing, China). SW480 cells were infected subcutaneously into each side of the back of every mouse for tumorigenesis experiments. The size of tumor was then detected by measuring the length (L) along with width (W) of the tumor with calipers every 3 days, and the tumor volume (V) was calculated by using the formula V = 1/2 × L × W [16].

### HE staining

The tumor tissue sections were dewaxed. The sections were then added to distilled water, and stained with an aqueous solution of hematoxylin for several minutes. The Colors of each section were separated in acid water and ammonia for a few seconds. The sections were rinsed under running water for 1 hour and rinsed with the addition of distilled water for a while. Then, the sections were dehydrated in 70% and 90% alcohol for 10 min each and stained with alcohol eosin staining solution for 3 minutes. The stained sections were dehydrated by pure alcohol and cleared with xylene [17]. The transparent portion was dropped with neutral gum and covered with a coverslip.

### Scratch wound assay

The cell migration capacity was measured by scratch wound assay. We scratched on the monolayers with sterile pipette tips (200 μL) and rinsed off the natant and separated cells with serum-free medium while the treated SW480 and HT29 cells were forming confluent monolayers in the wells of the 6-well plate [18]. Lastly, we took images with a reverse fluorescence microscope at 0 h and 48 h respectively [19]. For each image, we chose three locations to detect the width of the scratch at random and then averaged the results.

### Transwell

Inserts pre-coated with matrigel was used to perform Transwell so that the migration and invasion ability of SW480 and HT29 cell could be tested. Next, the suspension of 4 × 10^5^ cells was added to the supraventricular and the culture medium to the lower chamber in the migration experiment, while the lower chamber was added with a layer of culture medium and a layer of matrigel in the invasion experiment. After 48 hours, the non-invasive cells on the upper side of the membrane were cleaned up and the cells locating on the other side outside the membrane were treated with cold methanol (−20°C) and then dried in air [20]. Finally, the cells were stained with crystal violet and counted with an inverted microscope. The experiment was repeated three times.

### Western blotting

The total protein was extracted from SW480 cells with modified RIPA buffer solution. The proteins with equal amounts were split on 10% polyacrylamide gels, and then transferred to polyvinylidene fluoride membranes, which were put into the primary antibody *KIF18A* (Abcam, ab72417, 1:2000), *PTEN* (Abcam, ab170941, 1:1000), *p-PI3K* (Abcam, ab182651, 1:500), *p-Akt* (Abcam, ab38449, 1:1000), *MMP2* (Abcam, ab92536, 1:1000), *MMP9* (Abcam, ab76003, 1:1000) and anti-*GAPDH* (Abeam, ab128915, 1:10000) at 4°C overnight after blocked with 5% BSA. After that, we incubated the membranes with horseradish peroxidase-conjugated secondary antibodies and observed them with an ECL chemiluminescence system [21, 22]. Densitometric analysis was performed with a Bio-Rad image detection system and Quantity One software (Bio-Rad, Hercules, CA, USA). Each experiment was repeated in triplicate for statistical analysis.

### Statistical analysis

The statistical analyses were performed using GraphPad Prism 9.0 software. Comparisons among multiple sets of samples were performed with Studen’s *t*-test or one-way ANOVA with post-hoc testing (Dunnett multiple comparison). If *P* value is less than 0.05, the data is of statistical significance. Data is presented as mean ± standard deviations. Each experiment is performed in triplicate.

## RESULTS

### Decreased expression of *KIF18A* has something to do with the malignancy of colorectal cancer

**Figure 1:**
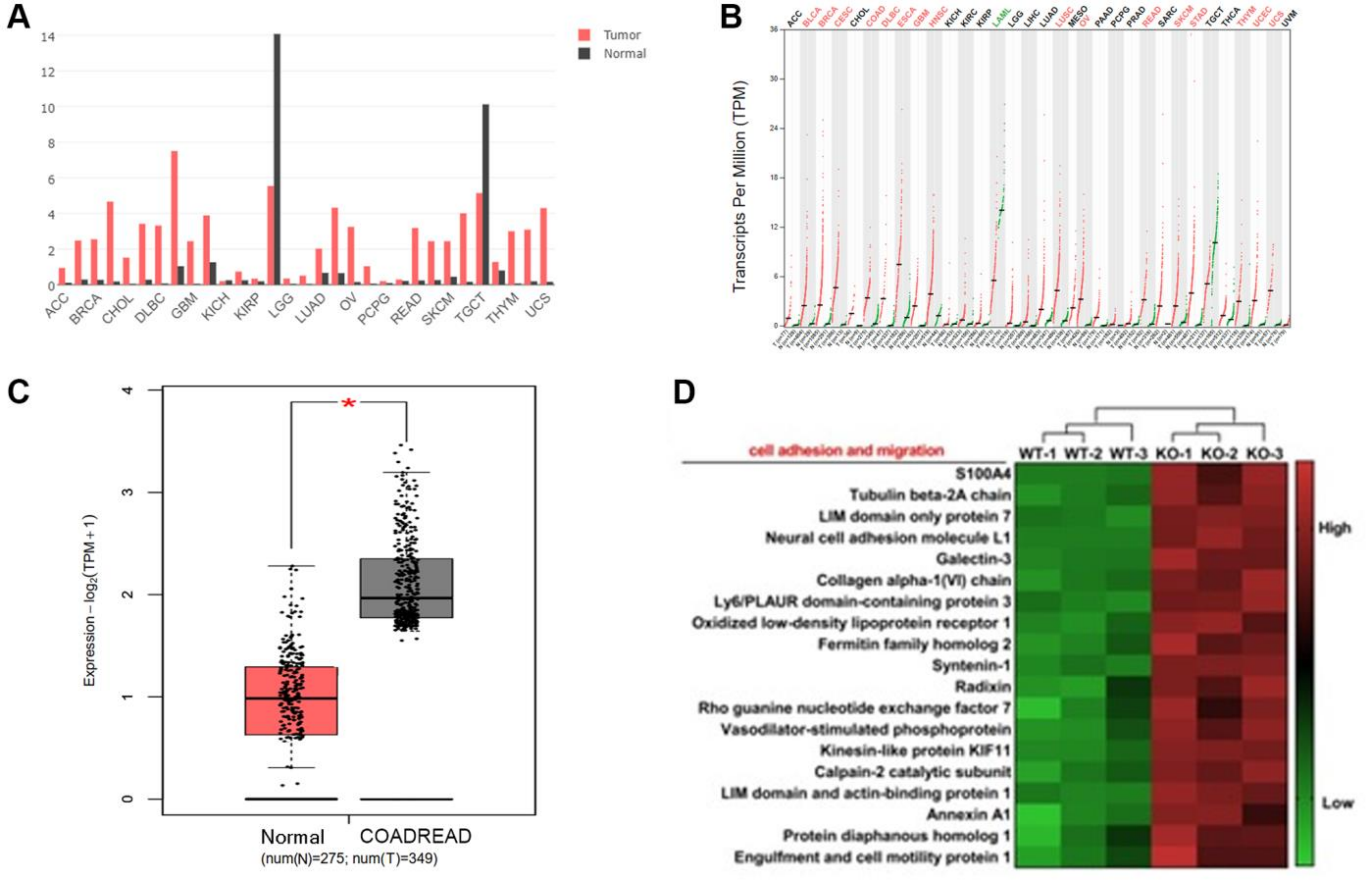
**The expression of *KIF18A* in colorectal cancer tissues.** (**A**, **B**) The transcriptional level (FPKM value) of *KIF18A* in pan-cancer tissues from the TCGA database. (Abbreviations: ACC: Adrenocortical Cancer; BRCA: Breast Invasive Carcinoma (Breast Cancer); CHOL: Cholangiocarcinoma (Bile Duct Cancer); DLBC: Diffuse Large B-Cell Lymphoma; GMB: Glioblastoma Multiforme (Brain Cancer); KICH: Kidney Chromophobe; KIRP: Kidney Renal Papillary Cell Carcinoma; LGG: Lower Grade Glioma; LUAD: Lung Adenocarcinoma; OV: Ovarian Serous Cystadenocarcinoma (Ovarian Cancer); PCPG: Pheochromocytoma and Paraganglioma; READ: Rectum Adenocarcinoma; SKCM: Skin Cutaneous Melanoma; TGCT: Testicular Germ Cell Tumors; THYM: Thymoma; UCS: Uterine Carcinosarcoma)). (**C**) The transcriptional level (FPKM value) of *KIF18A* in CRC and normal samples from the TCGA database. (COADREAD: Colon and Rectal Cancer). (**D**) The heatmap of genes involved in the cell adhesion and migration in sh*KIF18A*-SW480 cells.

To evaluate the effect of *KIF18A* on CRC progression, the expression abundance of *KIF18A* in cancers was analysed according to the public data. The clinical data and the FPKM (fragments per kilobase of transcript per million mapped reads) matrices were downloaded from GEO (GEO 24572). Compared with normal tissue, the transcription abundance of *KIF18A* was promoted in most of the solid tumour tissues, including breast cancer, bladder cancer, colorectal cancer etc. (Figure 1A, 1B). In colorectal cancer, it was obvious that the expression of *KIF18A* was promoted in tumour tissues (275 vs. 349, *P* = 0.017) (Figure 1C). Differences in gene expression between the sh*KIF18A*-SW480 and vector-SW480 cell lines (shown in Figure 1D) were compared by the transcriptome analysis.

### *KIF18A* can promote the progression of CRC

**Figure 2:**
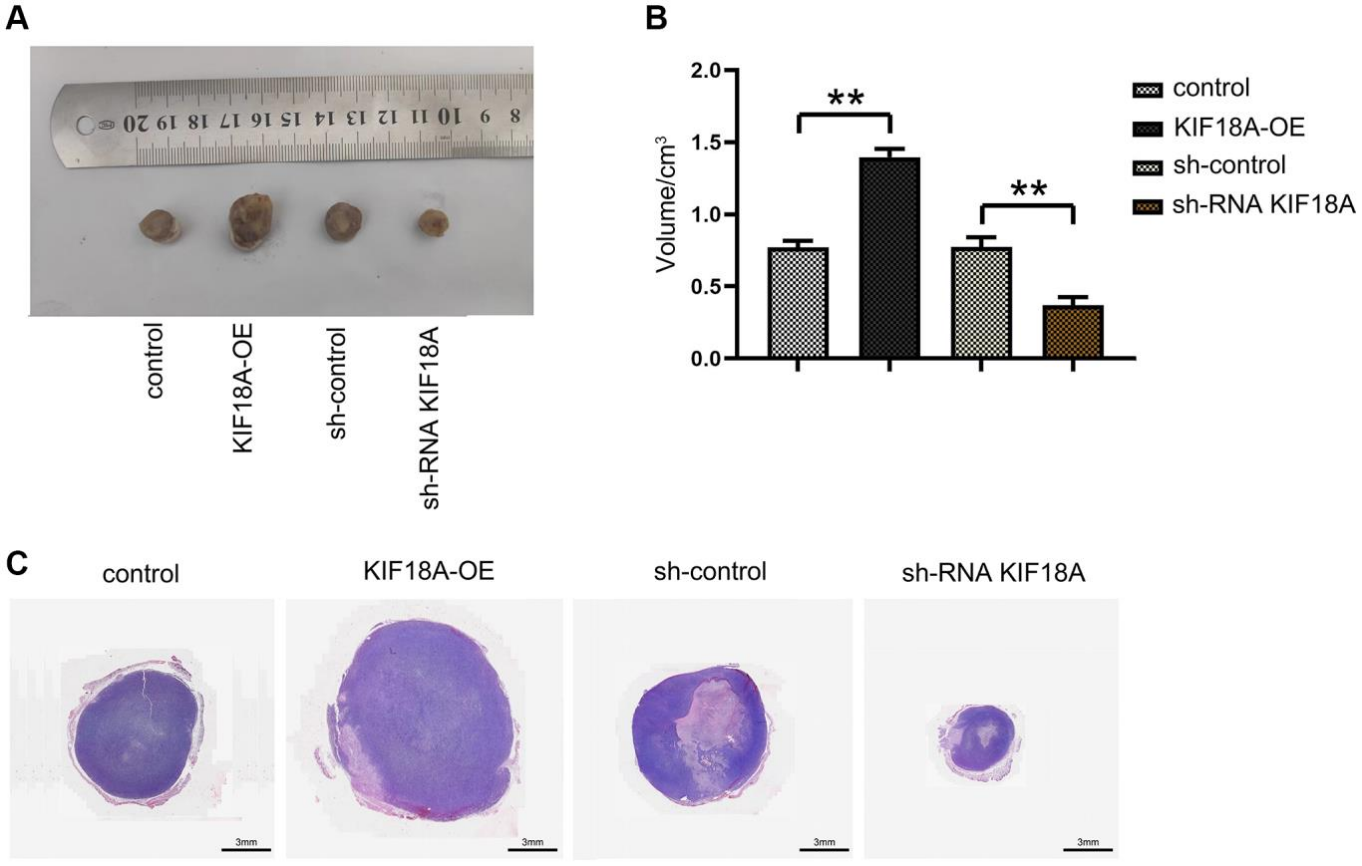
**Effect of *KIF18A* on the development of CRC.** (**A**) Diagrams of tumorigenesis in nude mice in sh-control group, sh-RNA *KIF18A*group, control group, *KIF18A*-OE group. (**B**) Statistics of tumor volume in nude mice in sh-control group, sh-RNA *KIF18A* group, control group, *KIF18A*-OE group. (**C**) Plots of HE staining results (^**^p < 0.01; *N* = 6).

For further investigate the effect of *KIF18A*, we transfected SW480 cell lines with sh*KIF18A* lentivirus and *KIF18A* overexpressing lentivirus. After transfecting, we injected them subcutaneously into nude mice to measure the size of tumors. It was shown that the size of tumors in the sh-RNA *KIF18A* group was distinctly smaller than those in the sh-control group. We also found that the size of tumors in the *KIF18A*-OE group was significantly larger than the control group. This result was then verified by HE staining (Figure 2). What mentioned above suggests that *KIF18A* can promote the progression of CRC.

### *KIF18A* activates the *PI3K*/*Akt* signaling pathway by targeting *PTEN to* affect the expression of MMPs

In order to determine whether *KIF18A* can regulate the expression of *PTEN* signaling pathway, we transfected sh*KIF18A* lentivirus and *KIF18A* overexpressing lentivirus into SW480 cells.

**Figure 3:**
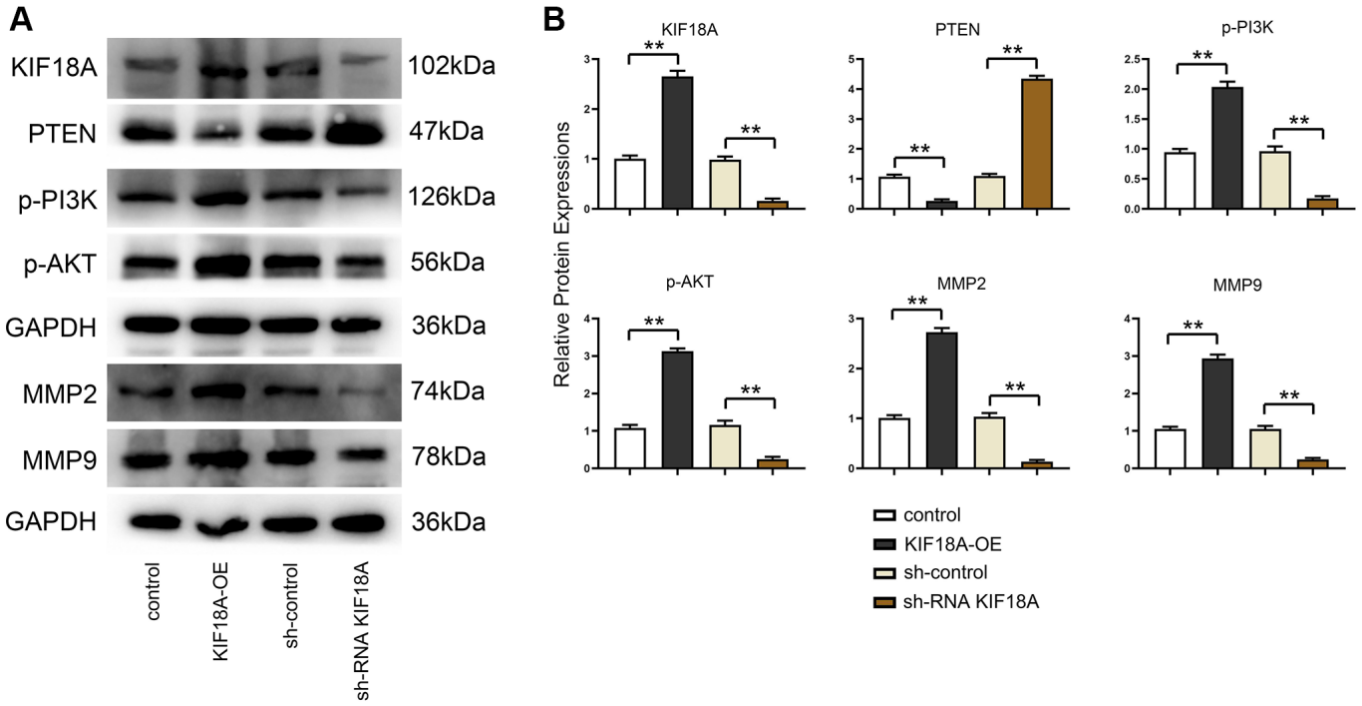
**Influence of *KIF18A* on *PTEN* and *PI3K*/*Akt* signaling pathway.** (**A**) Protein band diagrams of *KIF18A*, *PTEN*, *p-PI3K*, *p-Akt*, *MMP2* and *MMP9* in sh-control group, sh-RNA *KIF18A* group, control group, *KIF18A*-OE group. (**B**) Relative protein expressions of *KIF18A*, *PTEN*, *p-PI3K*, *p-Akt*, *MMP2* and *MMP9* in sh-control group, sh-RNA *KIF18A* group, control group, *KIF18A*-OE group (^**^p < 0.01; *N* = 3).

Western blotting results displayed that the relative protein expressions of *KIF18A*, *p-PI3K*, *p-Akt*, *MMP2* and *MMP9* were remarkably decreased and *PTEN* expression was signally risen in sh-RNA *KIF18A* group compared with the sh-control group, in the meantime, relative protein expressions of *KIF18A*, *p-PI3K*, *p-Akt*, *MMP2* and *MMP9* were going up and *PTEN* expression was dropping off in the *KIF18A*-OE group compared with the control group as plain as day (Figure 3).

### *KIF18A* can promote the migration and invasion of CRC cells

**Figure 4:**
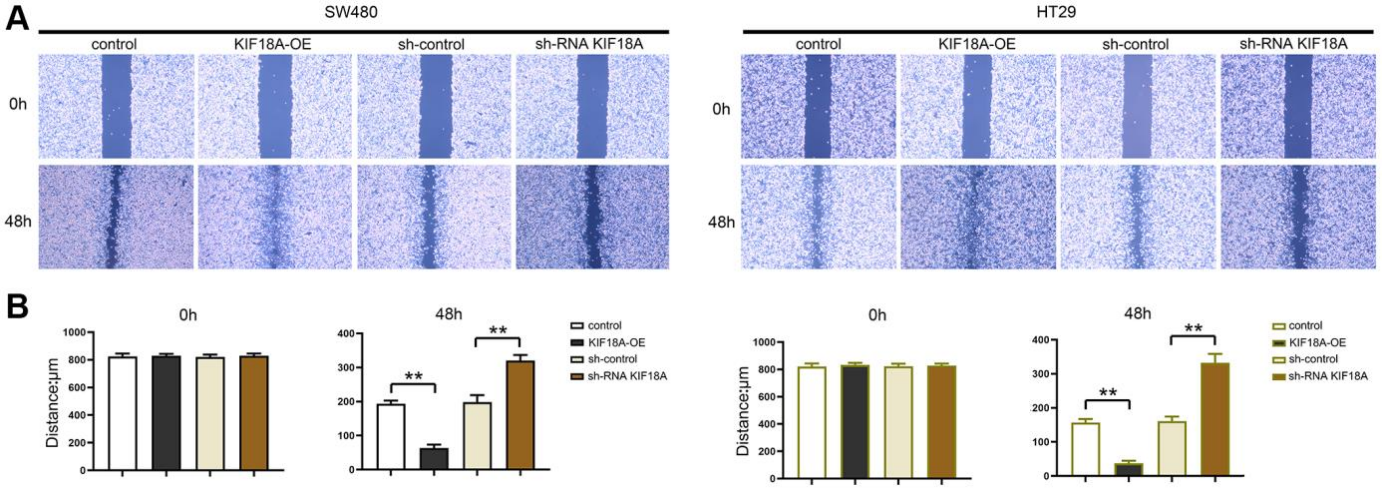
**Effect of *KIF18A* on CRC cells migration ability.** (**A**) Scratch wound diagrams at 0 h and 48 h in sh-control group, sh-RNA *KIF18A* group, control group, *KIF18A*-OE group. (**B**) Statistics of scratch-wound space at 0 h and 48 h in sh-control group, sh-RNA *KIF18A* group, control group, *KIF18A*-OE group (^**^p < 0.01; *N* = 3).

**Figure 5:**
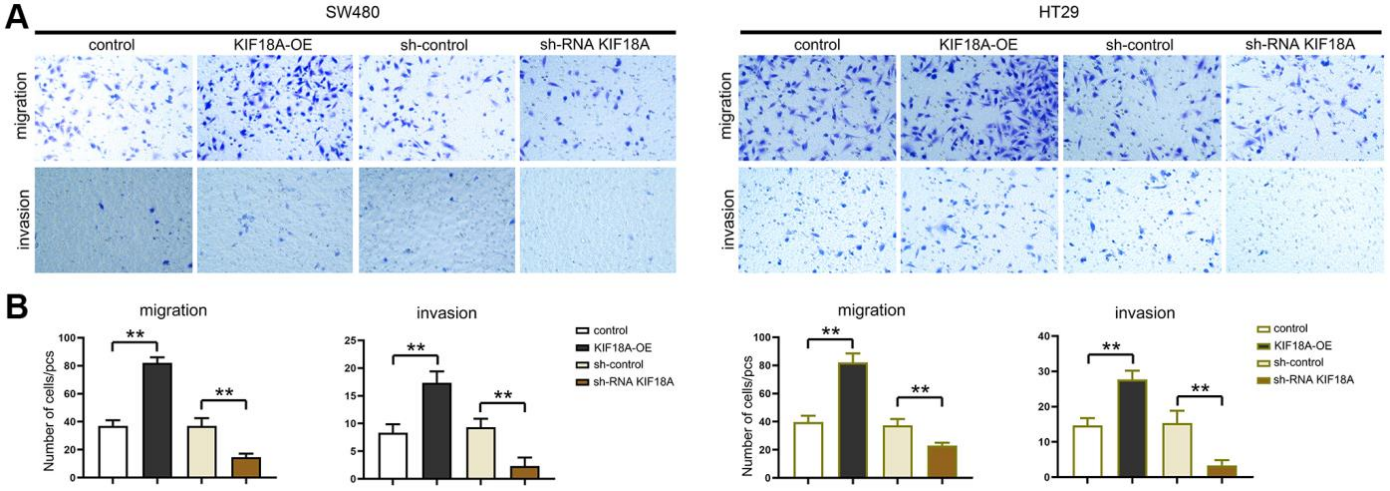
**Influence of *KIF18A* on CRC cells migration and invasion ability.** (**A**) Result diagrams of migration and invasion experiments in sh-control group, sh-RNA *KIF18A*group, control group, *KIF18A*-OE group. (**B**) Statistics of migration and invasion cells in sh-control group, sh-RNA *KIF18A* group, control group, *KIF18A*-OE group (^**^p < 0.01; *N* = 3).

We tested the migration and invasion ability of SW480 AND HT29 cells by scratch wound assay, and Transwell assay was conducted for researching the biological functions of *KIF18A* and CRC. Based on scratch wound assay, sh-RNA *KIF18A* group significantly increased the cell scratch space in relative to sh-control group at 48 h. Meanwhile, in *KIF18A*-OE group, the cell scratch space was observably reduced in contrast to control group (Figure 4). In the migration experiment, the results suggested that the amount of migrated cells in sh-RNA *KIF18A* group was remarkably cut down compared with sh-control group, and the number of migrated cells in *KIF18A*-OE group was obviously risen compared with the control group. Invasion tests showed that the number of invasive cells in sh-RNA *KIF18A* group was reduced obviously in comparison with sh-control group. The number of invading cells was significantly risen in the *KIF18A*-OE group compared with the control group (Figure 5). The above-mentioned results indicate that *KIF18A* can improve the migration and invasion ability of CRC cells.

### *KIF18A* can promote migration and invasion of CRC cells through targeting *PTEN* to activate *PI3K*/*Akt* signaling pathway

Western blotting results displayed that the relative protein expressions of *KIF18A*, *p-PI3K*, *p-Akt*, *MMP2* and *MMP9* were remarkably decreased and *PTEN* expression was signally risen in sh-RNA *KIF18A* group compared with sh-control group (Figure 5).

**Figure 6:**
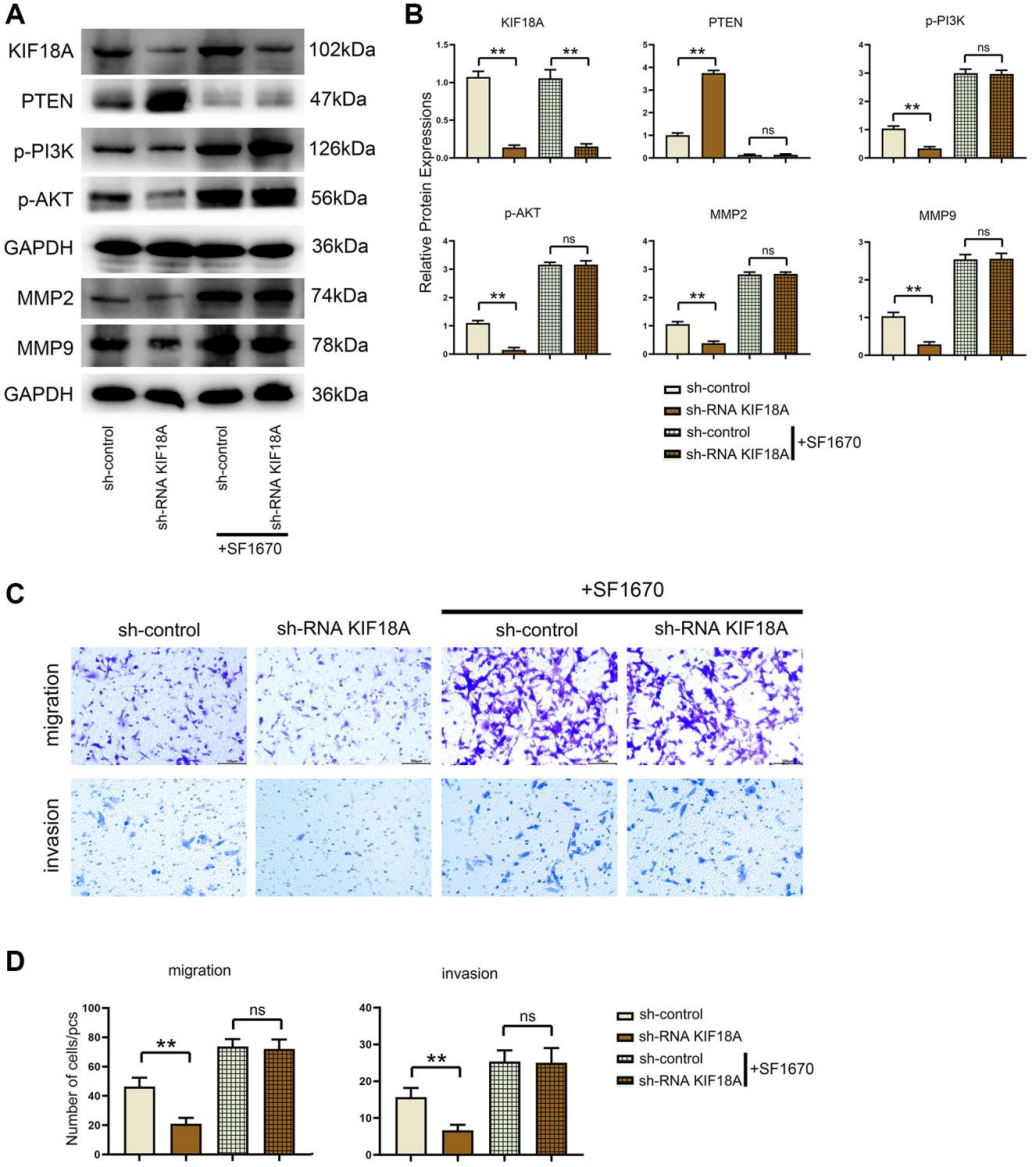
**Influences of *PTEN* inhibitor on *PTEN* and *PI3K*/*Akt* signaling pathways and migration and invasion of CRC cells in control and *KIF18A*-OE groups.** (**A**) Protein band diagrams of *PTEN*, *p-PI3K*, *p-Akt*, *MMP2* and *MMP9* in control group, *KIF18A*-OE group, control+SF1670 group and sh-RNA *KIF18A*+SF1670 group. (**B**) Relative protein expressions of *PTEN*, *p-PI3K*, *p-Akt*, *MMP2* and *MMP9* in control group, *KIF18A*-OE group, control+SF1670 group and sh-RNA *KIF18A*+SF1670 group. (**C**) Diagrams of migration and invasion experiments in control group, *KIF18A*-OE group, control+SF1670 group and sh-RNA *KIF18A*+SF1670 group. (**D**) Statistics of migration and invasion cells in control group, *KIF18A*-OE group, control+SF1670 group and sh-RNA *KIF18A*+SF1670 group (^**^p < 0.01; ns *p* > 0.05; *N* = 3).

**Figure 7:**
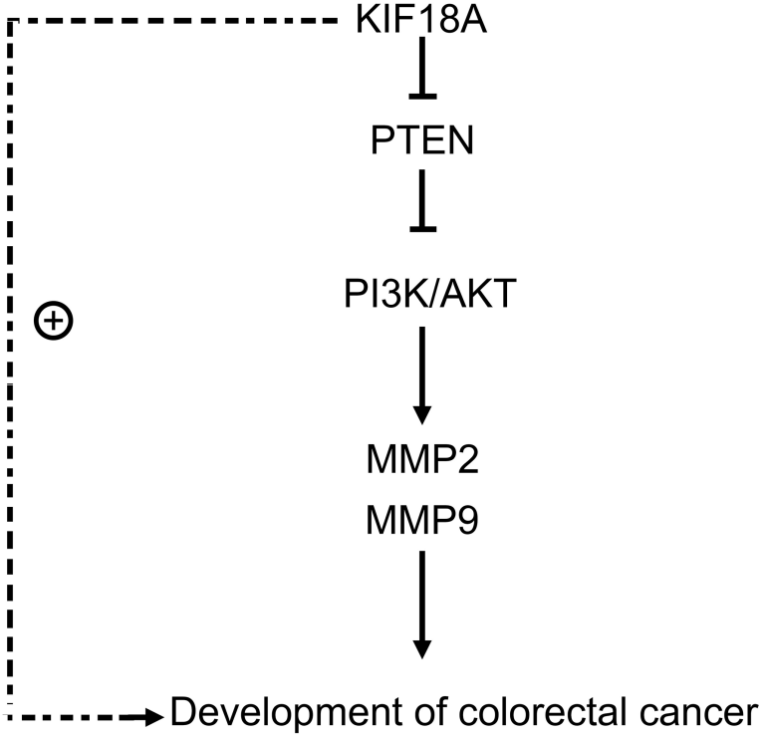
*KIF18A* can mediate *PTEN*, activate the *PI3K*/*Akt* signaling pathway, and promote the migration and invasion of CRC cells.

After adding the *PTEN* inhibitor SF1670, we found that there were not significant differences of the relative protein expressions of *PTEN*, *p-PI3K*, *p-Akt*, *MMP2* and *MMP9* between the sh-RNA *KIF18A*+SF1670 group and the control+SF1670 group. Besides, another finding was that the number of migration and invasion cells in sh-RNA *KIF18A* group was significantly lower than control group through Transwell. However, there was no significant difference in the number of migration and invasion cells between the control+SF1670 group and the sh-RNA *KIF18A*+SF1670 group (Figure 6), which indicated that *KIF18A* can increase the migration and invasion of CRC cells by inhibiting *PTEN* to promote the activation of *PI3K*/*Akt* signaling pathway (Figure 7).

## DISCUSSION

CRC is one of the most common malignancies, and its occurrence and development are related to multiple gene abnormalities and signal pathway dysregulation. Despite increasing progress in targeted therapy and immunotherapy, the overall survival of metastatic colorectal cancer is poor [23–25]. Studying the mechanism of metastasis is help to the development of new therapeutic targets. Research steps of this study are as follows. First, by analyzing the published data of the expression of TCGA and GEO, we found that the expression of *KIF18A* gene is increased in tumor tissues. What’s more, the expression of genes containing cell proliferation, cell cycle, cell adhesion and migration as well as EMT signaling pathway is up-regulated in *KIF18A* knockout cells, so we concluded that *KIF18A* may also directly or indirectly regulate the expression of genes including these four signal transduction. In previous studies, it was reported that *KIF18A* was involved in cell proliferation, migration as well as invasion of esophageal cancer [26] and chromosomally unstable tumor cells [27, 28]. However, we have never discovered the regulatory molecules and signaling pathways. In the research, we sought to uncover the molecules that interact with *KIF18A* and explore the effect of KIF18A on CRC and its mechanism of action.

*KIF18A*, as kinesin-like protein 18A, is part of kinesin superfamily. It plays a key role in microtubule dynamics as it helps to maintain proper chromosome arrangement. *KIF18A* consists of a motor domain (responsible for ATP-dependent movement along microtubules) and a coil domain, which promotes double protein interactions. It has been shown to interact with several proteins involved in mitotic spindle assembly. Additionally, KIF18 interacts with tumors to inhibit p53, exhibiting a potential regulatory role in cancer progression. Current studies have uncovered that *KIF18A* has a high expression in many human tumors, including CRC [29–32]. However, its functional molecular mechanisms in CRC have not been fully explored. In the study, we found that *PTEN* expression was damaged in *KIF18A* knockout cells, but increased in *KIF18A* overexpression cells, which indicated that *KIF18A* can target *PTEN* to regulate CRC cells and also promote the activation of *PI3K*/*Akt* signaling pathway, leading to the upregulation of *MMP2* and *MMP9* expression.

*PTEN* is an impressive tumor suppressor, and its full name is Phosphatase and tensin homolog. It is considered an important factor in regulating embryonic development, cell survival and metabolic balance. *PTEN* can restrict *Akt* (protein kinase) signaling pathway and prevent cell proliferation and migration through catalyzing the conversion of phosphatidylinositol 3,5-triphosphate (*PIP3*) to phosphatidylinositol 4-bisphosphate (*PIP2*) [33]. Besides, *PTEN* is also involved in the pathogenesis and process of type I diabetes mellitus, dyslipidemia and nervous system diseases through transmembrane protein and enzyme mechanisms. Studies have shown that abnormal expression of *PTEN* gene is closely connected with the occurrence and development of many cancers like prostate cancer, breast cancer, ovarian cancer and so on. Therefore, studying *PTEN* will be conducive to a deeper understanding of the pathogenesis of cancer and provide new mechanisms for CRC treatment. In this study, it was found that the migration and invasion of CRC cells can be inhibited if we knockout *KIF18A*, and *KIF18A* overexpression can promote the migration and invasion of CRC cells. We discovered that *KIF18A* regulates CRC cells by targeting *PTEN* through *PTEN* inhibitor SF1670.

These findings show the important influence of *KIF18A* on the occurrence and development of colorectal cancer, providing new ideas for further exploring the pathogenesis of colorectal cancer.

## CONCLUSION

We found that *KIF18A* whose expression could be up-regulated with the increase of tumor volume as a tumor-promoting gene in colorectal cancer. Down-regulation of *KIF18A* in tumors can control *PTEN* expression and promote activation of *PI3K*/*Akt* signaling pathway. Moreover, inhibition of *KIF18A* can control CRC cell migration and invasion. Based on the above-mentioned results, we can conclude that *KIF18A* can mediate *PTEN* and motivate the activation of *PI3K*/*Akt* signaling pathway, resulting to the progression of CRC.
